# Community perspectives on scabies, impetigo and mass drug administration in Fiji: A qualitative study

**DOI:** 10.1371/journal.pntd.0008825

**Published:** 2020-12-04

**Authors:** Elke Mitchell, Stephen Bell, Li Jun Thean, Aalisha Sahukhan, Mike Kama, Aminiasi Koroivueti, John Kaldor, Andrew Steer, Lucia Romani

**Affiliations:** 1 Kirby Institute, UNSW Sydney, Sydney, New South Wales, Australia; 2 Centre for Social Research in Health, UNSW Sydney, Sydney, New South Wales, Australia; 3 Tropical Diseases Group, Murdoch Children’s Research Institute, Melbourne, Victoria, Australia; 4 Department of Paediatrics, University of Melbourne, Melbourne, Victoria, Australia; 5 Ministry of Health and Medical Services, Suva, Fiji; 6 Melbourne Children’s Global Health, Melbourne Children’s Campus, The Royal Children’s Hospital, Melbourne, Australia; Universidade Estadual de Campinas, BRAZIL

## Abstract

Scabies is endemic in Fiji and is a significant cause of morbidity. Little is known about the sociocultural beliefs and practices that affect the occurrence of scabies and impetigo, or community attitudes towards the strategy of mass drug administration that is emerging as a public health option for scabies and impetigo control in Fiji and other countries. Data were collected during semi-structured interviews with 33 community members in four locations in Fiji’s Northern Division. Thematic analysis examined participants’ lived experiences of scabies and impetigo; community knowledge and perceptions about scabies and impetigo aetiology and transmission; community-based treatment and prevention measures; and attitudes towards mass drug administration. Many indigenous Fijian (iTaukei) participants noted extensive and ongoing experience of scabies and impetigo among children in their families and communities, but only one participant of Indian descent (Indo-Fijian) identified personal childhood experience of scabies. Scabies and impetigo were perceived as diseases affecting children, impacting on school attendance and families’ quality of sleep. Awareness of scabies and impetigo was considerable, but there were major misconceptions around disease causation and transmission. Traditional remedies were preferred for scabies treatment, followed by biomedicines provided by local health centres and hospitals. Treatment of close household contacts was not prioritised. Attitudes towards mass drug administration to control scabies were mostly positive, although some concerns were noted about adverse effects and hesitation to participate in the planned scabies elimination programme. Findings from this first study to document perspectives and experiences related to scabies and impetigo and their management in the Asia Pacific region illustrate that a community-centred approach to scabies and impetigo is needed for the success of control efforts in Fiji, and most likely in other affected countries. This includes community-based health promotion messaging on the social dynamics of scabies transmission, and a campaign of education and community engagement prior to mass drug administration.

## Author summary

Scabies is a skin disease causing discomfort from severe itchiness. It can lead to secondary bacterial infection of the skin (impetigo) that can in turn lead to systemic complications, including septicaemia, kidney disease and rheumatic heart disease. It can also cause sleep disorders and a reduced quality of life. Recently added to the World Health Organization list of neglected tropical diseases, scabies is estimated to affect 150 million people globally each year and is endemic in many Pacific Island countries, including Fiji. We aimed to increase understanding of community beliefs and practices that affect the occurrence of scabies and impetigo, and assess community attitudes towards the use of mass drug administration for scabies and impetigo control in Fiji. The impact of scabies and impetigo on participants’ quality of life included poor sleep quality, school absenteeism and social isolation, especially among children. Participants had awareness of scabies and impetigo, however, misconceptions around the cause and the ways in which these diseases were transmitted between individuals were common. Participants often reported choosing traditional medical remedies to treat scabies; treatment at health centres was mainly sought for secondary skin infections. Attitudes towards mass drug administration were positive, although some concerns regarding adverse effects were noted. Improved strategies, including a community-centred response, are needed for the success of control efforts in Fiji.

## Introduction

Scabies is a major public health problem in many low and middle income countries and a significant cause of morbidity [1,2]. It is a skin disease caused by infestation with *Sarcoptes scabiei* mites, which is estimated to affect 150 million people globally each year [2] and is endemic in many Pacific Island countries [2–4]. People with scabies often suffer discomfort from severe itchiness and sleep disorders [5,6]. In addition, scabies can lead to secondary bacterial infections of the skin (impetigo), which can in turn result in systemic complications including septicaemia, kidney disease and possibly rheumatic heart disease [2]. Scabies is transmitted through close personal contact with an infected person and can spread throughout households, schools, healthcare and other settings where people live or socialise and there are opportunities for skin contact [6,7]. Interlinked factors associated with scabies prevalence include low socioeconomic status, overcrowding, tropical climate and limited access to treatment [1,6,8,9]. In recognition of the burden and impact of scabies globally as well as the potential of new strategies for its control, scabies was recently added to the World Health Organization’s list of neglected tropical diseases [10].

Fiji has among the highest prevalence of scabies reported in the world [2,4,8,11], particularly in the Northern Division [4]. A national survey conducted in Fiji of over 10,000 residents reported an estimated national scabies prevalence of 18.5%, highest in the Northern Division (28.5%) [4]. Prevalence was highest in children aged five to nine years (43.7%). The prevalence of impetigo was 19.6%, highest in children aged five to nine years (34.2%) [4]. The prevalence of scabies and impetigo was twice as high in the iTaukei (Indigenous) Fijian than Indo-Fijian populations.

Standard treatment of scabies in endemic countries is often ineffective in reducing disease prevalence, even when treatment is extended to household or family contacts, possibly due to repeat infestations from untreated community members [8]. Mass drug administration (MDA) with topical permethrin or oral ivermectin treatments delivered to whole communities has been shown to be a safe and effective control strategy for scabies [3,12–15]. Extensive experience with the MDA strategy for the control of other neglected tropical diseases has demonstrated the importance of community support to ensure that high levels of treatment adherence are achieved [16–18].

Qualitative research provides insight into sociocultural practices, norms and beliefs that shape people’s understanding and management of health issues, and their response to public health initiatives such as MDA [19–21]. It is therefore a crucial underpinning to the development of control and health promotion programs. However there has so far been little qualitative research of this kind related to scabies and impetigo [16], and none in Fiji or other endemic countries in the Asia Pacific Region.

Drawing on data collected in 2019 during qualitative interviews with community members from four communities in the Northern Division of Fiji, this paper addresses this knowledge gap. Its aim is to document experience of scabies and impetigo, the sociocultural beliefs and practices that affect the occurrence of scabies and impetigo, and community attitudes towards the strategy of MDA for scabies and impetigo control.

## Methods

### Ethical statement

Ethical approval for this study was gained from the Fiji National Research Ethics Committee [2018.38.NOR] and the Royal Children’s Hospital Melbourne Human Research Ethics Committee [38020]. All participants were provided with a participant information sheet and consent form which the researcher explained to participants and responded to any questions, and which participants signed. Participation in the study was voluntary and confidential. Pseudonyms are used in reporting the data.

### Study setting

Located in the South Pacific, Fiji is made up of over 300 islands with a population of 884,887 in 2017, comprising two main ethnicities, iTaukei and Indo-Fijians [22]. Fiji is ranked 92 out of 189 countries in the United Nations Human Development Index and a gross national income per capita of $US8,324 in 2017 [23]. Life expectancy at birth is 73 years for women and 67 years for men [24]. English is used widely in Fiji and is the principal language of government, education and business. The Northern Division of Fiji, which includes Fiji’s second largest island, Vanua Levu, has a population of 131,914 [22] and includes the main towns of Labasa and Savusavu as well as smaller settlements and villages.

### Qualitative study design

This qualitative study was conducted in the context of a community intervention trial (known as BIG SHIFT) designed to assess the impact of ivermectin MDA on the control of scabies and its serious complications across the whole population of the Northern Division of Fiji. An interpretive research approach—which enables access to subjective, socially-situated, ‘emic’ or insider understandings and perceptions of health issues [25]–was adopted to achieve the study aims.

### Sampling and recruitment

Participants were residents of two villages and two settlements on Vanua Levu. The study was conducted in May 2019 during the BIG SHIFT pre-intervention baseline survey, for which 16 sentinel villages/settlements were randomly selected for a baseline skin survey, conducted by skin assessment teams which visited sites over a two-week period. The lead qualitative investigator (EM) joined two of the teams over a seven day period to conduct qualitative interviews with community members participating in the baseline skin survey.

Purposive sampling techniques [26] were used to recruit participants based on ethnicity (iTaukei and Indo-Fijian), gender and location (village and settlement). Due to the higher burden of disease among the iTaukei population (especially among children) and the gendered division of labour in Fiji (where women are primarily responsible for childcare and domestic work) we recruited a larger number of iTaukei and female participants into the study. This was done to gain more in-depth insight into child and family experiences of scabies and impetigo, and prevention and management of both diseases within households. Recruitment occurred during and after the skin assessments, with community members invited by a study nurse during the assessment or by the lead investigator once the skin assessment was completed. Participants were eligible if they were aged 18 years or older, lived in one of the sentinel villages/settlements and were able to provide informed consent.

### Data collection

Semi-structured discussion guides were used to explore participants’ experiences of scabies and impetigo within the family; impact of scabies and impetigo on daily life; knowledge of scabies and impetigo disease causation; preventative measures and health-seeking practices; and attitudes towards MDA. The development of these guides was informed by a socio-ecological model of health promotion [27] to examine personal (e.g. knowledge, perception of health), interpersonal (e.g. family), institutional (e.g. health services) and societal (e.g. policies, cultural norms) influences on participants’ perspectives, experiences and management of scabies and impetigo. Interviews lasted 20–45 minutes and were conducted in English by the lead investigator, who had prior experience in conducting qualitative health research in Fiji.

### Data analysis

Interviews were audiotaped and transcribed verbatim. Data were input into NVivo 11 and coded thematically using deductive and inductive techniques [26] by the lead investigator. Deductive coding examined personal, interpersonal, institutional and societal level influences on participants’ knowledge, perceptions, experiences and management of scabies and impetigo. Further inductive analysis was conducted to identify specific issues discussed by interviewees within each theme.

## Results

### Characteristics of participants

In total, 33 community members (21 iTaukei and 12 Indo-Fijian) participated in the study. Ages ranged from 19–68 years with a median of 41.5 years (Table 1). Most (26/33) participants were female, reflecting availability during the day when skin assessments predominantly took place. Household composition varied between 1–13 occupants; the median number of occupants in iTaukei households (seven) was higher than Indo-Fijian households (four), reflecting the typical living arrangements of the two groups in Vanua Levu.

**Table 1:** Characteristics of participants.

Participant characteristics	No. participants
*Physical location*	
Village	18
Settlement	15
*Ethnicity*	
iTaukei	21
Indo-Fijian	12
*Gender*	
Female	26
Male	7
*Age group*	
19 years and under	1
20–29 years old	5
30–39 years old	8
40–49 years old	9
50 years and over	9
Unknown	1
*Household composition*	
1–4 occupants	10
5–8 occupants	19
9–13 occupants	4

### Lived experiences of scabies and impetigo

Many iTaukei participants reported extensive current or previous experiences of scabies and impetigo in their households and communities, especially among children, with intense itching, skin lesions and fever reported as common disease manifestations for both diseases. With regard to scabies in the family, typical responses included, “Mostly it [scabies] affects only the children” (Illisapeci, iTaukei female, 33 years, village) and, “Only the children have this [scabies]” (Alesi, iTaukei female, 44 years, village). A few participants cited previous experience of scabies in older adults in the family:

“My Dad, he had it [scabies] last two months and he went to the hospital and he’s been treated… One day he was just sitting and he was saying to us ‘My fingers, between my fingers, it’s itchy’ and he was scratching it”.

(Lomani, iTaukei female, 31 years, village)

“Sometimes we [myself and my wife] had it on our hands… but not now”.

(Mikaele, iTaukei male, 68 years, village)

Many iTaukei participants discussed reoccurring scabies within their household:

“My son [has scabies]. I have very good hygiene at home but then he still gets these skin diseases. Itchy and all. We use the creams, but it keeps coming back and I don’t understand why”.

(Naomi, iTaukei female, 25 years, village)

Interviewer: You mentioned that your baby and yourself and your husband have some itching at the moment, *milamila* [itching]?

Participant: *Io* [yes]

Interviewer: How long have you had the itching for?

Participant: Two months

Interviewer: Have you had this before, or is this the first time?

Participant: We have had it before. (Litia, iTaukei female, 21 years, village)

Impetigo was less commonly discussed, but some iTaukei participants had a child or knew of a child in the community who had a current or previous case of impetigo.

“This [impetigo] is with my son… [He has had it] since last year… He was a baby, you know the diaper? Because I was not staying with him, because I was with my aunt, sometimes when I come if he has it, I take it off [the diaper]. He keep on scratching, gets really worse. Then he keeps on scratching it, and that’s why I think it gets [infected].”

(Elenoa, 32 years, iTaukei female, village)

In contrast to iTaukei participants, there were limited reported experiences of scabies and no reports of impetigo among Indo-Fijian participants. All but two Indo-Fijian participants stated they had never experienced scabies or impetigo in their family, with typical responses being, “No, we don’t have this in our family” (Bhadrak, Indo-Fijian male, 41 years, settlement) and, “In my family no, lucky” (Pravati, Indo-Fijian female, 68 years, settlement). One man disclosed experiencing scabies as a child, stating, “I had this when I was 12 [years old]. It used to itch a lot. I got it from playing outside” (Naksh, Indo-Fijian male, 46 years old, settlement). One woman said that her daughter had scabies symptoms at the time of research, including a rash and intense itching at night, but did not believe she had scabies:

“My daughter has some itching on [her] arms and legs. She is very itchy at night and it keeps her awake. I took her to the doctor, and he gave her some cream and said to use it on her whole body, but it hasn’t helped… I don’t think it is that disease [scabies]”.

(Akshi, Indo-Fijian female, 42 years, settlement)

#### Impact on daily life

Most iTaukei participants reported that excessive itching from scabies and impetigo resulted in poor sleep for children and mothers who often cared for children at night.

“I avoid her [daughter] from scratching it [impetigo], but I was with her the whole night and day to avoid [it] from getting worse, because if she scratches it, the sore will get bigger”.

(Lomani, iTaukei female 31 years, village)

“I have to stay up with him [son]… I have sleepless nights. And I have to scratch him and be up [with him]”.

(Unasi, iTaukei female 26 years, settlement)

A few iTaukei participants reported that their children experienced embarrassment, teasing and shame in primary school when they had severe cases of impetigo. As a coping mechanism, these children stopped interacting with peers to avoid these experiences or attempted to conceal the visible signs of impetigo through wrapping hands with material.

“The [children] get shy because they got this thing [impetigo] and people can point it out. I think they stay indoors. Because they move out there are more children, and this one [points to son] they tease”.

(Kala, iTaukei female, 34 years, village)

“Mostly they get embarrassed about it. My daughter she gets embarrassed and upset she misses the school…When they have this thing [impetigo], they say they always wrap their hands because they know that if their friends see obviously, they tease. Just to hide it”.

(Lomani, iTaukei female, 31 years, village)

School absenteeism due to scabies or impetigo was reported regularly, with children missing two or three days of school for scabies and up to a week or more for more serious cases of impetigo. Some iTaukei participants kept children at home when they had scabies or impetigo symptoms to reduce onward transmission. Others reported children being sent home from school by teachers or the school principal to prevent transmission, with advice to parents to keep children at home until they were treated.

“I think when they have this sickness [scabies] they should stay at home, otherwise it goes to school and that thing will be spread”.

(Ela, iTaukei female, 63 years, settlement)

“The schools don’t, they don’t let you take the sickness [scabies] inside the school. So, the students may [have] infected them, they have to stay at home”.

(Levani, iTaukei female, 39 years old, village)

### Knowledge and perceptions about scabies and impetigo aetiology and transmission

Overall awareness of scabies and impetigo among participants was widespread across the sample. Both iTaukei and Indo-Fijian participants perceived scabies and impetigo to be diseases experienced by children.

#### Local terminology

Participants used a range of local terms to refer to scabies and impetigo. For scabies, the most common was the Fijian word “*karokaro*” which was used to describe the papules that present on the skin of people infected with scabies. The Fijian word “*drimi*”–a term used for itchy fungal infections of the skin with blisters—was used by a few people to describe impetigo rather than ringworm. Local words used to describe the symptoms experienced by people infected with scabies included the Fijian word “*milamila*” and the Fiji-Hindi word “*khajuli*”, both of which translate to ‘itching’. *Milamila* was sometimes also used to describe impetigo.

#### Disease causation

Rather than reporting mites as the cause of scabies, participants’ perceptions about scabies causation were formed via lived experiences of these diseases in households and communities. As such, iTaukei participants reported more ideas on disease causation than Indo-Fijian participants.

Most iTaukei participants made strong associations between scabies and heavy rain and poor drainage near homes during the wet season. These environmental conditions resulted in a build-up of stagnant, dirty water as well as the contamination of nearby rivers which were frequently used by children as a place to bathe and play.

“It’s [village] in a very low-lying area. Whenever there is very heavy rain, we have big flood… That is why a lot of people you see have this sickness, these people, you see them with *milamila*. Because most of the people are having this *milamila* [due to] the flooding, because we don’t have proper drainage. When we have this very heavy rain you can see, most of the time it doesn’t flow… stagnant water”.

(Felise, iTaukei female, 47 years, village)

“They [children] always play in the dirty water… Most of the time, they get scabies here… [from] bathing in the river, dirty water… After it rains then they play in the dirty water”.

(Mikaele, iTaukei male, 68 years, village)

As result of children spending time outside, especially when environmental conditions were more extreme, scabies was also closely linked to children’s dirty clothing, and their limited bathing and hand washing practices.

“It is during this rainy weather. It just gets really muddy. I think it’s contributing to them [children getting scabies] because they go and play in the mud. They bring it inside, you know those kinds of dirty waters from the creek, from the ground, from the holes. They usually play with it, and when they come, they keep on scratching… and they don’t wash their bodies properly with the soap and water, things get stuck into their bodies”.

(Elenoa, iTaukei female, 32 years, village)

“They [children] play in the mud. Yeah and the thing appears [scabies] the days after, just because we have many dogs in the village. The shit of the dog, they go and play in the mud”

(Levani, iTaukei female, 39 years, village)

“I think because they [children] don’t bath properly at home. And like some of them are sick at home, and they [get] the itchiness, so that’s how they got it”.

(Unasi, iTaukei female, 26 years old, settlement)

Only a few iTaukei participants associated impetigo with excessive scratching and open scabies sores:

“Some they feel like scratching it and [they get] those swollen ones with the pus [impetigo]. In some with *milamila* the skin [becomes] infected, it starts to have like a water or something coming out of their skin”.

(Felise, iTaukei female, 47 years, village)

Instead, impetigo was perceived as a consequence of difficulties cleaning and drying clothing and bedding during prolonged periods of wet weather and flooding:

“I think the environment. Sometimes we Fijians, what we mostly do, we take all the beddings outside to dry it in the sun. I think if people don’t dry these beddings and all, people are, clothes are damp, and this is some of the causes of this [impetigo], very itchy.”

(Joni, iTaukei male, 50 years, village)

“This thing [impetigo], he [son] get it from last month. From the heavy rain, it started. It makes him itchy. It’s spreading.”

(Akanisi, iTaukei female, 23 years, village)

There was limited understanding of scabies causation among Indo-Fijian participants. The few Indo-Fijian participants who did discuss causation also linked scabies to poor personal hygiene, especially among children. No Indo-Fijian participants offered an explanation for the cause of impetigo.

#### Disease transmission

Most Indo-Fijian and iTaukei participants were unable to provide an explanation for how scabies and impetigo are transmitted, with typical responses being, “I’m not really sure” (Seruwaia, iTaukei female, 62 years, village) and, “I don’t know” (Edha, Indo-Fijian female, 21 years, settlement). Among those who did, two main transmission routes for scabies and impetigo were identified. The first was contact with items such as clothing, bedding, soap and utensils that were shared within the home.

“How is it spread? Normally in the same family, they start sharing the same clothes… [It spreads] among family members”

(Timoci, iTaukei male, 41 years, village)

“The soap used in the house. It passes [through] the soap. Maybe some get scabies and reuse that soap”

(Akanisi, iTaukei female, 23 years, village)

The second was through close contact with infected individuals, with a particular focus on children in home and school settings. This included transmission between children through hugging, holding hands, sleeping in the same bed and sharing clothes:

“It [scabies] can spread easily, because they [children] sleep together, or hug, playing yeah. It’s easily spread”.

(Kala, iTaukei female, 34 years, village)

“I think it [impetigo] spreads because it’s a bacteria, fungal infection. When they [children] hold hands, eat together, things get spread that way… Especially my kids, they are of the same height, same age group, all wear the same kind of clothes”.

(Elenoa, iTaukei female, 32 years, village)

No participants identified possible transmission between adults through sexual contact, nor between adults and children through breastfeeding or childcare.

### Treatment of scabies and impetigo

#### Traditional methods

Most iTaukei participants described use of traditional medical remedies to manage episodes of scabies in children and adults. This included the use of the Fiji medicine ‘*rewa*’–a technique passed down from previous generations and shared between women in villages or settlements—that is believed to have a curative effect. This medicine is the combination of coconut oil with leaves of medicinal plants (often described as ‘herbs’) found throughout Vanua Levu—including *makosoi*, *uci*, *moli karo*, *drala*, *botebotekoro*, and *dilo*–which is then applied onto the affected area of the skin and allowed to dry. Use of this medicine dries the skin and was reported to stop itching for 2–4 days at which point the mixture would be reapplied in order to prevent further itching.

“In the village, we have the medicine [*rewa*]. We get the herbs, when we bring the coconut oil, we cook it in the coconut oil, with herbs… We have certain kinds of herbs that we use, like our grandmothers, our grandparents they used too, and they passed it on, and we will keep passing it on… they just grow around here and are easy to find… The children, we bath them, clean them properly, the ones who are affected from them, who suffer from the *milamila*, scabies. And then we apply [*rewa*]”.

(Felise, Taukei female, 47 years, village)

“When they [children] have the bumps [scabies], then I would have the herbal medicine [*rewa*] and just massage it where the bumps are… [It] stops the itchiness, especially in the night, when they sleep”.

(Unasi, iTaukei female, 26 years, settlement)

“Usually we dry [leaves] in the fire and then we mix it with oil [to make *rewa*]… My hands are dry now, when I’m itchy I apply it, and then I’m not itchy… It stops, maybe 3 or 4 days, then I get itchy again”.

(Netani, iTaukei female, 19 years old, village)

*Rewa* was also occasionally used in mild cases of impetigo after the skin had been cleaned with warm, salty water. No participants disclosed that they or other adults used precautionary measures to prevent transmission when treating children or other family members with *rewa*.

#### Seeking medical care

Most iTaukei participants disclosed that they would use *rewa* as a first remedy to manage scabies and would only seek medical care for prolonged scabies cases and if the scabies rash turned into a secondary skin infection (impetigo).

“When you put it [*rewa*] at the right time, when it starts itching you put the oil, then the thing will finish. But when the thing is very itching, like that [impetigo] then it won’t work… Sometimes we put oil and it finish, but sometimes we go to hospital”.

(Levani, iTaukei female, 39 years, village)

“I think the first medical treatment we can give here, most of the people you know [use] the herbal medicines [*rewa*]… Only second what people do is go to the hospital”.

(Joni, iTaukei male, 50 years, village)

Only a few iTaukei participants reported going straight to the doctor or hospital when scabies first appeared.

“For my four children when I see them with this skin disease, we go straight to the health centre. The one that’s just in town… I explain everything, it’s either they get the injection [antibiotics], or they’re given the cream”.

(Lomani, iTaukei female, 31 years, village)

On seeking care at health centres or hospitals for scabies or impetigo, iTaukei participants reported that a nurse or doctor usually provided cream and /or gave their child an antibiotic injection to treat the secondary infection. Most participants said that the nurse directed use of cream for the infected children only and rarely advised other people (especially adults) in the family to also be treated.

“She [nurse] asked how many days, for the rashes. I told her one week and they gave her [daughter] antibiotics… They told me to bath her in saltwater”.

(Sereana, iTaukei female, 39 years, settlement)

Participant: I normally take him to the health centre… They just give some cream

Interviewer: When the nurse gave the cream, was it for your son only, or for others in the family as well?

Participant: No, only for the son, not for the family (Timoci, iTaukei male, 41 years, village)

Indo-Fijian participants reported seeking medical assistance as soon as skin issues occurred. When asked about treatment of hypothetical scabies or impetigo cases, Indo-Fijian participants typically responded, “I would go to the doctor or hospital if they [child] had this” (Pravati, Indo-Fijian female, 68 years, settlement).

### Preventing scabies and impetigo

iTaukei participants, especially women, reported using a range of strategies to prevent or reduce likelihood of onward transmission of scabies and impetigo. Prevention strategies included “boil [wash at high temperature] the clothes” (Felise, iTaukei female, 47 years, village), “take the bed, the mattress in the sun” (Levani, iTaukei female, 39 years, village), “wash it [skin] with the hot water and the salt” (Litia, iTaukei female, 21 years, village), “clean up the home and surroundings” (Timoci, iTaukei male, 41 years, village), “stop children playing in dirty areas” (Sereana, iTaukei female, 39 years, settlement) and “wash their [children’s] hands properly with soap and water” (Elenoa, iTaukei female, 32 years, village).

“The moment they get the sickness [scabies], I put their clothes, the clothes they’re wearing, I put it aside, they don’t wear it again. It’s what I do… I just wash the clothes. That’s important, they don’t wear them again today, that’s what I do”.

(Naomi, iTaukei female, 25 years, village)

Such preventative information was sometimes provided by nurses during appointments while children were treated for scabies or impetigo.

“They [nurses] usually ask what the cause the sickness, and I just explain to them. They give the advice to avoid going to those areas, playing in those muddy waters and just keep them [children] inside during the wet season”.

(Lomani, iTaukei female, 31 years, village)

In a village setting, management and prevention of scabies and impetigo was often a social, community-centred practice. iTaukei women reported sharing advice and information on treatment with *rewa* or medical care and measures to prevent onward transmission, and working closely together to manage scabies and impetigo at both household and village levels.

“In the community, we advise the child. Because the community’s a village. We [women] talk about it, we share it, if we can cure. If we can’t then we take them to the hospital.”

(Kala, iTaukei female, 34 years old, village)

“We went to the hospital with a woman [from the village]… with same disease as our children, she also has children, I think they had spread disease… I told her I think it’s our clothes because the weather, I think because it was no sunshine, for a time it was rainy, and no ventilation, I think that’s why it start because we don’t put our clothes in the sun, just hang it around here”.

(Naomi, iTaukei female, 25 years old, village)

“We [women] usually share information, how to mix herbal medicine [*rewa*], how to cure it”.

(Elenoa, iTaukei female, 32 years old, village)

Few iTaukei participants identified the social causes of scabies and impetigo—avoiding personal contact with an infected person—in discussions about prevention. Those that did reported close monitoring of other children in the village and stopped their children playing with children with scabies or impetigo.

“I just stop my children going beside him or her [another child with scabies]… until the child is better. Because it spread easily. If they stick together, body touch like that they can get it”.

(Kala, iTaukei female, 34 years, village)

“I always be watching my children… I always worry about them [other children], I always ask their mothers, what’s wrong with your children, take them to the hospital or do something with them… I have to protect my kids, that’s why I protect others. It’s very upsetting when you see kids like that, and they feel bad for getting it”.

(Ilisapeci, iTaukei female, 33 years, village)

### Attitudes towards MDA for scabies and impetigo control

The majority of participants reported they had not heard of the specific terminology “mass treatment” or “MDA”, with typical responses being, “I’m not sure” (Aadhira, Indo-Fijian female, 52 years, settlement), “No, this is the first time” (Lomani, iTaukei female, 31 years, village) and, “No never” (Bhadrak, Indo-Fijian male, 41 years, settlement). After explanation was provided regarding the planned scabies elimination programme, two reasons were given in support of MDA treatment approaches (i.e. familiarity with treatment approaches, importance of prevention), while three barriers to MDA were identified (i.e. a preference for Fiji medicine, concerns about possible side effects after taking unnecessary treatment, confusion about the treatment protocol).

#### MDA is a familiar treatment approach

Many iTaukei and Indo-Fijian participants recalled receiving medication for the treatment of lymphatic filariasis within the last five years. Responses such as, “We took the filariasis tablets, maybe two years ago” (Darika, Indo-Fijian female, 47 years, settlement) and, “Yes for mosquito, I think it was last year” (Ela, iTaukei female, 63 years old, settlement), suggested participants were familiar with the approach. MDA for lymphatic filariasis has been administered across Fiji for the past 18 years [28], and most participants saw no reason why this approach could not also be used for scabies. When asked about the upcoming scabies MDA, people responded with “It will be helpful… Just like the mosquito one, it will prevent it from spreading” (Vilisi, iTaukei female, 19 years, village) and, “it might stop the itchiness and prevent the scabies” (Unasi, iTaukei female, 26 years, settlement).

#### Prevention is important

Many Indo-Fijian participants viewed the elimination programme as a good precautionary measure, despite many not experiencing scabies or impetigo in the family with typical responses being, “It’s a good preventative. Prevention is better than cure” (Darika, Indo-Fijian female, 47 years, settlement). Similarly, when discussing MDA for treatment of endemic diseases in Fiji, common responses included, “Of course, we are into this [medicine] so happy to take” (Aadhira, Indo-Fijian female, 52 years, settlement).

Several iTaukei participants’ noted the importance of biomedical approaches to treating endemic health issues in their community and preventing future scabies and impetigo cases:

“It’s very important for us to take it. Because, Fijian’s, they know Fiji medicine, but this they should have [MDA]… because I know some disease come, we don’t have that in the past [scabies], but we have it now. That’s why we have to take the advices from doctors and nurse, that’s why we have to take it”.

(Naomi, iTaukei female, 25 years, village)

“If they [community members] take tablets [for scabies] it’s good for the village, because to stop this type of sickness, otherwise it spread in the village and we will be sick again… we have to take the tablets to cure this, got this sickness, we have to take the tablet”.

(Ela, iTaukei female, 63 years old, settlement)

Improved overall health and wellbeing of family members and the wider community, and the reduced time and cost associated with delivering treatment in community settings, were also noted by iTaukei participants as benefits of MDA:

“I just want a healthy and happy family. So, whatever can contribute for the better health of the family, I will always want it”.

(Elenoa, iTaukei female, 32 years, village)

“They [community] will be very happy [to participate in MDA]. You will supply some creams and some antibiotics [tablets], saves them time to go to the hospital… saves money”.

(Unasi, iTaukei female, 26 years old, settlement)

#### Preference for Fiji medicine over biomedical treatment

A few iTaukei participants reported that they would not be willing to take tablets for scabies and impetigo due to a preference for traditional medical remedies over biomedical treatments. Typical responses included, “give them *rewa* [instead]” (Sairusi, iTaukei male, 39 years, village) and, “I don’t know why they try to cure this kind, better to just use *rewa*” (Seruwaia, iTaukei female, 62 years, village).

#### Concern over side effects of unnecessary treatment

A few Indo-Fijian participants expressed apprehension about the side effects of taking medication that is not needed. Concerns included the possibility of allergic reaction to the medication provided during MDA, with questions such as, “What happens if I or someone [else] reacts to the medication? Could we get sick?” (Basanti, Indo-Fijian female, 42 years, settlement). The potential for negative health consequences from taking tablets to treat scabies and impetigo if family members were not currently infected was also raised in comments such as, “If we don’t have scabies and we take tablets will it affect us?” (Edha, Indo-Fijian female, 21 years, settlement). Others questioned if it was alright to participate in the MDA if they had underlying health issues, such as heart disease or diabetes. For instance, Pravati (Indo-Fijian female, 68 years, settlement) asked, “I have a heart issue, is it OK to take [the medication]?”

#### Confusion about treatment protocol

There was confusion among a few iTaukei and Indo-Fijian participants as to whether everyone needed to take medication during the elimination programme, or just those with scabies or impetigo symptoms, with questions like, “If we don’t have the sickness, we take the tablets too?” (Ela, iTaukei female, 63 years, settlement). This highlights the importance of ensuring pre-MDA campaign community engagement efforts reach every member of the community.

“Some they just don’t get it… some [question] ‘What is the tablets for’, you know like that. I think if you give them the tablet you have to give them information, proper information. What is tablet for. Especially in this community level… [because] some they don’t feel like taking the tablets, it depends on the mindset… So before giving all those tablets, we need more information. Come right down to a community level and show the pictures. Taking this tablet and [explain] this is why”.

(Felise, iTaukei female, 47 years, village)

A few Indo-Fijian participants reported they would not be comfortable taking tablets for scabies if they were not symptomatic at the time of drug distribution:

“We don’t take the scabies tablets because we are not like this [don’t have scabies]. If we have skin diseases yes, if not it’s OK [don’t need to take the tablets].”

(Naksh, Indo-Fijian male, 46 years, settlement)

“If everything is OK [no scabies infection] then I won’t take the medication”

(Vibha, Indo-Fijian female, 48 years, settlement)

## Discussion

Our findings demonstrate diverse understandings of and responses to scabies and impetigo among two ethnic groups—iTaukei and Indo-Fijian—in four communities in Vanua Levu, Fiji. To our knowledge, this is the first qualitative study that explores community perspectives, experiences and management of scabies and impetigo, and attitudes towards MDA for scabies in the Asia-Pacific region, and one of the first in the world. As such our findings provide important insight into the improvement of scabies prevention, health promotion, management and control.

Experiences of scabies and impetigo were reported in children more than adults, and among iTaukei compared with Indo-Fijian participants. iTaukei participants described reoccurring experiences of scabies and impetigo in their families and communities, especially among children, while only two Indo-Fijian participants reported ever having experienced scabies-like symptoms within their families. There is limited understanding of why prevalence of scabies and impetigo is higher in the iTaukei population, but it has been suggested that this is due to living circumstances such as more children per family and more occupants within a single household [8]. Our findings support this assessment, with iTaukei participants in our study having on average seven people per household (ranging from 3–13 people per household) compared to four among Indo-Fijian participants (ranging from 1–8 people per household).

Awareness of scabies and impetigo was widespread and local terms for scabies–*karokaro*, *milamila* and *khajuli*–were well known among most participants. Disease manifestations for scabies and impetigo included intense itching of the skin, skin lesions and fever. The effects of experiencing scabies and impetigo were noted on daily routine and quality of life among iTaukei participants. Discussions centred on the negative impacts of scabies and impetigo for children, including school absenteeism and sleep disturbance due to intense itching at night [5,6,16]. Children’s experiences of embarrassment and stigma related to scabies and impetigo at school were highlighted by a few iTaukei participants. Previous research in Guinea-Bissau [16] and Brazil [29] has also highlighted the impact of scabies on children’s experiences of shame, stigma and societal isolation.

Understandings of disease causation were shaped by lived experiences of scabies and impetigo within households and communities among iTaukei participants, and through hearsay among Indo-Fijian participants. No participants reported that scabies is caused by mites; instead, associations were made between incidence of scabies and environmental factors (e.g. heavy rain and poor drainage), personal hygiene practices (e.g. dirty clothes and poor hygiene habits) and close contacts, affecting children in particular [16]. Only a few iTaukei participants accurately identified that impetigo can be caused by excessive scratching, instead making associations between impetigo and persistent wet weather and the inability to dry clothes and bedding materials.

There was a profound lack of discussion about the interpersonal, social and gendered dimensions of scabies and impetigo transmission and prevention of onward transmission. Only a few iTaukei participants associated scabies transmission with skin-to-skin contact among children in home and school settings, while no participants identified women’s roles in breastfeeding or caring for children as possible transmission routes, nor sexual contact. Accordingly, only a few iTaukei participants identified avoiding personal contact with an affected person as a preventative measure, and again focussed on transmission between children. Given higher prevalence of scabies and impetigo in iTaukei children compared to adults [4], it is not surprising that participants focused on children’s experiences during interviews. However, scabies and impetigo are experienced within families and communities, and as such, adults potentially do play a role in scabies transmission even if this role is not well recognised.

Management of scabies and impetigo occurred in household, community and health service settings. Among iTaukei participants, at home and in communities, women largely took responsibility for these issues. With a focus on children’s cleanliness and hygiene [16], iTaukei women worked together to clean clothes, dry bedding and furniture outside in the sun, clean the home and surrounding areas in the village, stop children playing in dirty areas and ensure that children washed their hands with soap. Women were also responsible for preparing and using traditional medical remedies to treat scabies in children with symptoms.

With regard to treatment, traditional medical remedies were reported as the preferred choice to treat scabies among iTaukei participants [16] with many only seeking medical care at health centres or hospitals if a scabies rash turned into a secondary skin infection. In contrast, all Indo-Fijian participants said they sought medical assistance in the first instance whenever a skin issue occurred and indicated they would do the same when scabies or impetigo first presented. Notably, on seeking medical care for scabies rash, iTaukei participants reported that nurses typically advised the use of cream on affected children only and rarely directed its use on all close family contacts (especially adults). While participants were unable to specifically name the type of cream provide during medical visits, permethrin cream is the first line treatment for scabies in Fiji and benzathine penicillin injections or oral cotrimoxazole for five days are used to treat impetigo [30], indicating participants were likely talking about permethrin cream. Findings suggest that national treatment guidelines—that specify treatment of all close contacts [30]–are not always followed at a clinical level and treatment of adults is not prioritised in the family context.

Awareness of MDA was limited but most iTaukei and Indo-Fijian participants indicated that they would participate in the upcoming scabies elimination programme by ingesting the drugs. This may be attributed to their knowledge on scabies and impetigo, familiarity with MDA as an approach to control lymphatic filariasis in Fiji, as well as a desire to treat current scabies cases and prevent future infestation. A few Indo-Fijian participants expressed concern about medication side effects, as well as perceived negative health consequences associated with taking medication for scabies if they were not symptomatic at the time of the elimination programme. A few iTaukei participants also expressed some hesitation to take part in the MDA, citing a preference for traditional medicines over biomedical treatment.

### Study limitations

When interpreting our findings, it is important to note that participants were recruited from urban, peri-urban and rural villages/settlements within 5 to 20 km from the main town of Labasa. Consequently, they were within easy access of health services and pharmacies where they could seek treatment for scabies and impetigo. Community members living in more remote locations in Vanua Levu and throughout Fiji, where access to healthcare is more limited, may have different perceptions, experiences and health-seeking behaviours regarding scabies and impetigo. In addition, no Indo-Fijian participants in the study reported issues with scabies or impetigo within their households, which may reflect the recruitment of Indo-Fijian participants from higher socio-economic backgrounds, residing in less overcrowded dwellings in scattered settlements near the town of Labasa. Thus, the experiences of less affluent Indo-Fijian households, including those who experiences scabies and impetigo within their families are missing from our study. Future research would benefit from focusing on community members residing in locations where access to healthcare is more limited as well as research with members of the Indo-Fijian community with lower socio-economic status households.

### Policy and program implications

Our findings point to several areas for action to address scabies control in Fiji. First, enhanced community-based health promotion is required to raise awareness of what scabies is, how it is transmitted, and the actions that can be taken to prevent and treat interpersonal transmission within households, schools and community settings. While a focus on interactions between children is essential, health promotion messages might also focus on the roles of parents, grandparents, teachers and other community adults involved in caring for children. It would be worth exploring how women, other village leaders and teachers who are involved in existing community, household and school-centred scabies management practices and networks can lead new community-based health promotion strategies.

Second, the use of traditional medicines was favoured as a first line treatment for scabies and to a lesser extent impetigo among iTaukei participants. In order to support broader scabies and impetigo control efforts in Fiji, reducing the gap between traditional knowledge systems to improve health and biomedicines is essential, as has been noted in efforts to address lymphatic filariasis in Papua New Guinea [18]. However, even when seeking biomedical care, our findings suggest health workers in health centres or hospitals may not always include the treatment of close contacts, especially adults. Barriers among healthcare workers to providing adequate care among cases and their close contacts need to be explored. Additional training and education of healthcare workers to ensure national guidelines are adhered to regarding the treatment of close household contacts in scabies cases is needed. It is also important to ensure adequate availability of permethrin at health facilities to enable dispensation of treatment for close contacts.

Finally, our findings indicate the need to increase community knowledge about MDA and associated benefits for control of scabies and impetigo, including the need for high levels of community adherence. Given the importance of community support to the success of elimination programmes [16–18], ensuring pre-MDA campaign community education and engagement efforts in Fiji has a wide reach is essential.

## Conclusion

Our findings provide novel insights into the diverse community perspectives, experiences and responses to scabies and impetigo among two ethnic groups—iTaukei and Indo-Fijian—in Fiji. These insights will prove valuable in designing and improving control strategies for scabies and impetigo. Findings highlight several areas for action, including the need for community-centred approaches to scabies and impetigo control efforts, roll out of community-based health promotion messaging on the social dynamics of scabies transmission, and pre-MDA campaign education and community engagement in order to ensure successful control efforts in Fiji.

## References

[1] Heukelbach J, Feldmeier H. Scabies. The Lancet. 2006;367(9524):1767–74. 10.1016/S0140-6736(06)68772-2 16731272

[2] Romani L, Steer AC, Whitfeld MJ, Kaldor JM. Prevalence of scabies and impetigo worldwide: a systematic review. The Lancet Infectious Diseases. 2015;15(8):960–7. 10.1016/S1473-3099(15)00132-2 26088526

[3] Romani L, Whitfeld MJ, Koroivueta J, Kama M, Wand H, Tikoduadua L, et al Mass Drug Administration for Scabies Control in a Population with Endemic Disease. New England Journal of Medicine. 2015;373(24):2305–13. 10.1056/NEJMoa1500987 . 26650152

[4] Romani L, Koroivueta J, Steer AC, Kama M, Kaldor JM, Wand H, et al Scabies and Impetigo Prevalence and Risk Factors in Fiji: A National Survey. PLOS Neglected Tropical Diseases. 2015;9(3):e0003452 10.1371/journal.pntd.0003452 25738499 PMC4349858

[5] Jackson A, Heukelbach J, Filho AFdS, Campelo EdB Júnior, Feldmeier H. Clinical features and associated morbidity of scabies in a rural community in Alagoas, Brazil. Tropical Medicine & International Health. 2007;12(4):493–502. 10.1111/j.1365-3156.2006.01809.x 17445140

[6] Karim SA, Anwar KS, Khan MAH, Mollah MAH, Nahar N, Rahman HEMR, et al Socio-demographic characteristics of children infested with scabies in densely populated communities of residential madrashas (Islamic education institutes) in Dhaka, Bangladesh. Public Health. 2007;121(12):923–34. 10.1016/j.puhe.2006.10.019. 17884117

[7] Bouvresse S, Chosidow O. Scabies in healthcare settings. Current Opinion in Infectious Diseases. 2010;23(2):111–8. 10.1097/QCO.0b013e328336821b 20075729

[8] Romani L, Whitfeld MJ, Koroivueta J, Kama M, Wand H, Tikoduadua L, et al The Epidemiology of Scabies and Impetigo in Relation to Demographic and Residential Characteristics: Baseline Findings from the Skin Health Intervention Fiji Trial. The American journal of tropical medicine and hygiene. 2017;97(3):845–50. Epub 07/17. 10.4269/ajtmh.16-0753 . 28722612 PMC5590570

[9] Feldmeier H, Jackson A, Ariza L, Lins Calheiros CM, de Lima Soares V, Oliveira FA, et al The epidemiology of scabies in an impoverished community in rural Brazil: Presence and severity of disease are associated with poor living conditions and illiteracy. Journal of the American Academy of Dermatology. 2009;60(3):436–43. 10.1016/j.jaad.2008.11.005. 19064303

[10] WHO. Neglected tropical diseases Geneva: World Health Organization; 2019 [cited 2019 18th December]. https://www.who.int/neglected_diseases/diseases/en/.

[11] WHO. Epidemiology and Management of Common Skin Diseases in Children in Developing Countries. Geneva: 2005.

[12] Marks M, Toloka H, Baker C, Kositz C, Asugeni J, Puiahi E, et al Randomized Trial of Community Treatment With Azithromycin and Ivermectin Mass Drug Administration for Control of Scabies and Impetigo. Clin Infect Dis. 2019;68(6):927–33. 10.1093/cid/ciy574 . 29985978 PMC6399435

[13] Lawrence G, Leafasia J, Sheridan J, Hills S, Wate J, Wate C, et al Control of scabies, skin sores and haematuria in children in the Solomon Islands: another role for ivermectin. Bull World Health Organ. 2005;83(1):34–42. Epub 01/21. . 15682247 PMC2623469

[14] Romani L, Marks M, Sokana O, Nasi T, Kamoriki B, Cordell B, et al Efficacy of mass drug administration with ivermectin for control of scabies and impetigo, with coadministration of azithromycin: a single-arm community intervention trial. The Lancet Infectious Diseases. 2019;19(5):510–8. 10.1016/S1473-3099(18)30790-4 30956111 PMC6483975

[15] Mohammed KA, Deb RM, Stanton MC, Molyneux DH. Soil transmitted helminths and scabies in Zanzibar, Tanzania following mass drug administration for lymphatic filariasis—a rapid assessment methodology to assess impact. Parasit Vectors. 2012;5:299-. 10.1186/1756-3305-5-299 . 23259465 PMC3543323

[16] Lopes MJ, Silva ETd, Ca J, Gonçalves A, Rodrigues A, Mandjuba C, et al Perceptions, attitudes and practices towards scabies in communities on the Bijagós Islands, Guinea-Bissau. bioRxiv. 2019:574327 10.1101/574327 PMC6974396 31722016

[17] Marks M, Kwakye-Maclean C, Doherty R, Adwere P, Aziz Abdulai A, Duah F, et al Knowledge, attitudes and practices towards yaws and yaws-like skin disease in Ghana. PLOS Neglected Tropical Diseases. 2017;11(7):e0005820 10.1371/journal.pntd.0005820 28759580 PMC5552343

[18] Wynd S, Carron J, Selve B, Leggat PA, Melrose W, Durrheim DN. Qualitative analysis of the impact of a lymphatic filariasis elimination programme using mass drug administration on Misima Island, Papua New Guinea. Filaria J. 2007;6:1-. 10.1186/1475-2883-6-1 . 17196113 PMC1770912

[19] Mitchell E, Bennett LR. Young women’s perceptions and experiences of sexual risk in Suva, Fiji. Culture, Health & Sexuality. 2020;22(5):504–19. 10.1080/13691058.2019.1614669 31144607

[20] Mitchell E, Lazuardi E, Rowe E, Anintya I, Wirawan DN, Wisaksana R, et al Barriers and Enablers to HIV Care Among Waria (Transgender Women) in Indonesia: A Qualitative Study. AIDS Education and Prevention. 2019;31(6):538–52. 10.1521/aeap.2019.31.6.538 31815531

[21] Bell S, Kennedy E, Black K, Vallely A, Vallely L, Mola G, et al Youth-centred research to help prevent and mitigate the adverse health and social impacts of pregnancy amongst young Papua New Guineans. Reproductive Health Matters. 2018;26(54):5–12. 10.1080/09688080.2018.1512297 30257613

[22] Fiji Bureau of Statistics. 2017 Population and Housing Census. Suva: Fiji Bureau of Statistics, 2018.

[23] United Nations Development Programme. Human Development Reports: Table 1. Human Development Index and its components: United Nations Development Programme; 2019 [cited 2019 5th November]. http://hdr.undp.org/en/composite/HDI.

[24] World Health Organization. Countries: Fiji: World Health Organization; 2019 [cited 2019 5th November]. https://www.who.int/countries/fji/en/.

[25] Bell S, Aggleton P. Interpretative and ethnographic perspectives: Alternative approaches to monitoring and evaluation practice In: Bell S, Aggleton P, editors. Monitoring and evaluation in health and social development. London: Routledge; 2016 p. 1–14.

[26] Patton MQ. Qualitative research and evaluation methods 3rd ed Thousand Oaks, CA: Sage; 2002.

[27] McLeroy KR, Bibeau D, Steckler A, Glanz K. An Ecological Perspective on Health Promotion Programs. Health Education Quarterly. 1988;15(4):351–77. 10.1177/109019818801500401 3068205

[28] Ichimori K, Graves PM. Overview of PacELF-the Pacific Programme for the Elimination of Lymphatic Filariasis. Trop Med Health. 2017;45:34-. 10.1186/s41182-017-0075-4 . 29118654 PMC5664808

[29] Worth C, Heukelbach J, Fengler G, Walter B, Liesenfeld O, Feldmeier H. Impaired quality of life in adults and children with scabies from an impoverished community in Brazil. International Journal of Dermatology. 2012;51(3):275–82. 10.1111/j.1365-4632.2011.05017.x 22348561

[30] Ministry of Health and Medical Services. Fiji Guidelines for Sore Throat and Skin Disease: Diagnostic and Treatment Guidelines October 2018. Suva: Ministry of Health and Medical Services, 2018.

